# Complications in Patients with Acute Myocardial Infarction Supported with Extracorporeal Membrane Oxygenation

**DOI:** 10.3390/jcm9030839

**Published:** 2020-03-19

**Authors:** Saraschandra Vallabhajosyula, Malcolm R. Bell, Gurpreet S. Sandhu, Allan S. Jaffe, David R. Holmes, Gregory W. Barsness

**Affiliations:** Department of Cardiovascular Medicine, Mayo Clinic, Rochester, MN 55905, USA; Bell.Malcolm@mayo.edu (M.R.B.); Sandhu.Gurpreet@mayo.edu (G.S.S.); Jaffe.Allan@mayo.edu (A.S.J.); Holmes.David@mayo.edu (D.R.H.J.); Barsness.Gregory@mayo.edu (G.W.B.)

**Keywords:** acute myocardial infarction, extracorporeal membrane oxygenation, complications, vascular, hemorrhage

## Abstract

Background: There are limited data on complications in acute myocardial infarction (AMI) admissions receiving extracorporeal membrane oxygenation (ECMO). Methods: Adult (>18 years) admissions with AMI receiving ECMO support were identified from the National Inpatient Sample database between 2000 and 2016. Complications were classified as vascular, lower limb amputation, hematologic, and neurologic. Outcomes of interest included temporal trends, in-hospital mortality, hospitalization costs, and length of stay. Results: In this 17-year period, in ~10 million AMI admissions, ECMO support was used in 4608 admissions (<0.01%)—mean age 59.5 ± 11.0 years, 75.7% men, 58.9% white race. Median time to ECMO placement was 1 (interquartile range [IQR] 0–3) day. Complications were noted in 2571 (55.8%) admissions—vascular 6.1%, lower limb amputations 1.1%, hematologic 49.3%, and neurologic 9.9%. There was a steady increase in overall complications during the study period (21.1% in 2000 vs. 70.5% in 2016). The cohort with complications, compared to those without complications, had comparable adjusted in-hospital mortality (60.7% vs. 54.0%; adjusted odds ratio 0.89 [95% confidence interval 0.77–1.02]; *p =* 0.10) but longer median hospital stay (12 [IQR 5–24] vs. 7 [IQR 3–21] days), higher median hospitalization costs ($458,954 [IQR 260,522–737,871] vs. 302,255 [IQR 173,033–623,660]), fewer discharges to home (14.7% vs. 17.9%), and higher discharges to skilled nursing facilities (44.1% vs. 33.9%) (all *p* < 0.001). Conclusions: Over half of all AMI admissions receiving ECMO support develop one or more severe complications. Complications were associated with higher resource utilization during and after the index hospitalization.

## 1. Introduction

Extracorporeal membrane oxygenation (ECMO) is increasingly used in acute cardiovascular care for the management of acute myocardial infarction (AMI) complicated by cardiac arrest, cardiogenic shock and complications from cardiac interventional procedures [1,2,3,4,5,6,7,8,9,10]. ECMO provides cardiac output support of nearly 3–5 L, biventricular support and respiratory support, thereby assisting with critical cardiorespiratory support in extreme situations [11]. Despite limited data and the absence of randomized controlled trials, ECMO has been widely accepted in clinical practice [2,3,4,5,6,7,8]. Despite the stated benefits from ECMO therapy, these acutely ill patients receiving ECMO support for AMI continue to experience high in-hospital mortality [7]. As compared to the intra-aortic balloon pump (IABP), the newer mechanical circulatory devices, such as the percutaneous left ventricular assist device (pLVAD) and ECMO, are associated with higher rates of complications [1]. There are limited contemporary data on complications in AMI patients receiving ECMO support [1]. As compared to other populations receiving ECMO support such as acute decompensated heart failure and post-cardiotomy cardiogenic shock, AMI patients are unique in the urgency related to insertion, often receive peripheral cannulation, may benefit from left ventricular unloading, may have concomitant cardiac arrest, and constitute >80% of cardiogenic shock etiology. Therefore, it is important to understand the complications and outcomes associated with ECMO in AMI. Using a 17-year national database, we sought to systematically evaluate a contemporary United States population.

## 2. Material and Methods

The National (Nationwide) Inpatient Sample (NIS) is the largest all-payer database of hospital inpatient stays in the United States. NIS contains discharge data from a 20% stratified sample of community hospitals and is a part of the Healthcare Cost and Utilization Project (HCUP), sponsored by the Agency for Healthcare Research and Quality [12]. Information regarding each discharge includes patient demographics, primary payer, hospital characteristics, principal diagnosis, up to 24 secondary diagnoses, and procedural diagnoses. These data are available to other authors via the HCUP-NIS database with the Agency for Healthcare Research and Quality.

During the period between 1 January 2000 through 31 December 2016, a retrospective cohort of admissions from the HCUP-NIS with a primary diagnosis of AMI (International Classification of Disease-9 Clinical Modification [ICD-9CM] 410.x; International Classification of Disease-10 Clinical Modification [ICD-10CM] I21.x–22.x) receiving ECMO support (ICD-9CM 39.65; ICD-10CM 5A15223) were identified consistent with prior literature [7]. Deyo’s modification of Charlson Comorbidity Index was used to identify co-morbid diseases and prior methodology was used to identify cardiac and non-cardiac procedures [2,3,7,8,11,13,14,15,16,17,18,19,20,21,22,23]. We identified relevant complications and categorized them as (a) vascular complications—arterial injury, acquired arterio-venous fistula, and vascular complications requiring surgery; (b) lower limb amputation; (c) hematologic—post-operative hemorrhage, hemolytic anemia, thrombocytopenia, and blood transfusion; and (d) neurologic—ischemic or hemorrhagic stroke (Supplementary Table S1). The primary outcome was the temporal trend in complications in and secondary outcomes included in-hospital mortality, hospitalization costs and length of stay in AMI admissions supported with ECMO.

### Statistical Analysis

As recommended by HCUP-NIS, survey procedures using discharge weights provided with HCUP-NIS database were used to generate national estimates. Using the trend weights provided by the HCUP-NIS, samples from 2000–2011 were re-weighted to adjust for the 2012 HCUP-NIS re-design. Chi-square and *t*-tests were used to compare categorical and continuous variables respectively. The inherent restrictions of the HCUP-NIS database related to research design, data interpretation, and data analysis were reviewed and addressed. Pertinent considerations include not assessing individual hospital-level volumes (due to changes to sampling design detailed above), treating each entry as an ‘admission’ as opposed to individual patients, restricting the study details to inpatient factors since the HCUP-NIS does not include outpatient data, and limiting administrative codes to those previously validated and used for similar studies. Univariable analysis for trends, predictors and outcomes was performed and were represented as odds ratio with 95% confidence interval. Multivariable logistic regression analysis incorporates age, sex, race, primary payer, comorbidity, hospital characteristics, acute organ failure, cardiogenic shock, cardiac arrest, type of AMI, cardiac, and non-cardiac procedures. For the multivariable modeling, regression analysis with purposeful selection of statistically (liberal threshold of *p* < 0.20 in univariate analysis) and clinically relevant variables was conducted. Two-tailed *p* < 0.05 was considered statistically significant. All statistical analyses were performed using SPSS v25.0 (IBM Corp, Armonk, NY, USA).

## 3. Results

During this 17-year period, there were over 10 million AMI admissions, of which 4608 (<0.01%) received ECMO support. ST-segment-elevation AMI, cardiogenic shock and cardiac arrest were noted in 66.4%, 80.9% and 45.6%, respectively. Mean age was 59.5 ± 11.0 years, 3490 (75.7%) were men, 2716 (58.9%) were of white race, and mean Charlson Comorbidity Index was 4.1 ± 2.0. Of these, over 85% were admitted to large teaching hospitals. Acute respiratory, renal, hepatic, hematologic, and neurologic dysfunction was noted in 63.2%, 63.6%, 32.3%, 37.4%, and 28.1% respectively. Coronary angiography, percutaneous coronary intervention, and pulmonary artery/right heart catheterization were used in 52.2%, 40.5%, and 23.1%, respectively. Concomitant IABP and pLVAD were used in 49.2% and 13.5%, respectively. Median time to ECMO placement was 1 day (interquartile range [IQR] 0–3).

**Figure 1:**
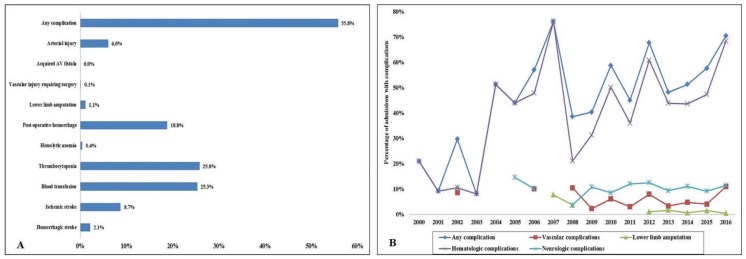
Complications in acute myocardial infarction admissions supported by extracorporeal membrane oxygenation. (**A**) Relative percentages of in-hospital complications across the various complication categories; (**B**): 17-year temporal trends of in-hospital complications; *p* < 0.001 for trend. AV: arterio-venous.

Complications were noted in 2571 (55.8%) admissions—vascular complications 6.1%, lower limb amputations 1.1%, hematologic complications 49.3%, and neurologic complications in 9.9% (Figure 1A). Thrombocytopenia, need for blood transfusion, and post-operative hemorrhage were the most common complications. The 17-year temporal trends showed a steady increase in complications, primarily due to an increase in hematologic complications (Figure 1B). In a multivariable logistic regression analysis, non-white race, non-Medicare insurance, higher comorbidity, admission to an urban hospital, admission to a medium and large-sized hospital, non-cardiac organ failure, and non-cardiac organ support were associated with development of complications (Supplementary Table S2).

**Figure 2:**
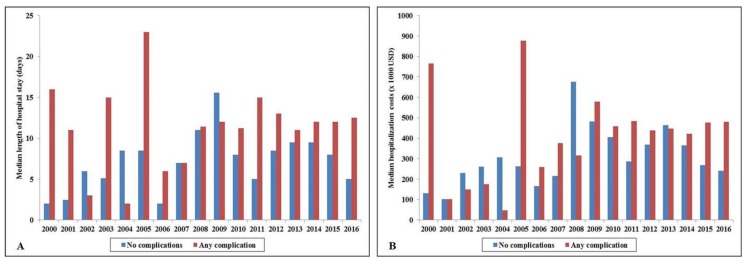
In-hospital resource utilization in acute myocardial infarction admissions supported by extracorporeal membrane oxygenation with and without complications. (**A**) Seventeen-year temporal trends of median length of stay in admissions with and without complications; *p* < 0.001 for trend; (**B**): 17-year temporal trends of median hospitalization costs in admissions with and without complications; *p* < 0.001 for trend. USD: United States Dollars.

The cohort with complications, compared to those without, had higher in-hospital mortality (60.7% vs. 54.0%; unadjusted odds ratio 1.31 (95% confidence interval 1.17–1.48); *p* < 0.001). In a multivariable logistic regression analysis, presence of complications was not independently associated with higher in-hospital mortality in ECMO recipients (odds ratio 0.89 (95% confidence interval 0.77–1.02); *p* = 0.10) (Table 1). The cohort with complications had longer median hospital stay (12 (IQR 5–24) vs. 7 (IQR 3–21) days), higher median hospitalization costs ($458,954 (IQR 260,522–737,871) vs. 302,255 (IQR 173,033–623,660)), fewer discharges to home (14.7% vs. 17.9%), and higher discharges to skilled nursing facilities (44.1% vs. 33.9%) (all *p* < 0.001). The cohort with complications had longer hospitalization and higher hospitalization costs over the 17-year period (Figure 2A,B).

## 4. Discussion

In the largest study looking at complications from ECMO use in AMI, nearly 56% of admissions had ≥1 complication. Hematologic complications, specifically thrombocytopenia, which are needed for blood transfusion and post-operative hemorrhage, were the most commonly noted complications. During this 17-year period, there was a steady increase in complications in AMI admissions receiving ECMO support. The presence of complications was associated with greater resource utilization during and after hospitalization, but had comparable in-hospital mortality to ECMO recipients without complications.

In patients with AMI, ECMO is often used to support tenuous hemodynamics during cardiac arrest, cardiopulmonary resuscitation, cardiogenic shock, and high risk coronary intervention [1,2,3,4,5,6,7,8]. However, by virtue of its unique configuration, ECMO is associated with higher left ventricular afterload, greater vascular complications due to large bore access, limb ischemia due to lack of antegrade flow, greater clotting, and thrombocytopenia due to a complex circuit and higher risk of strokes due to variation in anticoagulation [1]. There are limited data on the complications in AMI patients supported with ECMO [1]. In this study, we report comparable rates of hemorrhage, blood transfusion, arterial injury and limb ischemia, but slightly higher rates of thrombocytopenia compared to prevalent literature [1]. Patients with AMI are unique in that they frequently receive urgent peripheral cannulation, and therefore, carry a higher risk of left ventricular stasis and thrombosis. We have previously reported on the concomitant use of IABP and pLVAD, but are unable to assess if these were placed for left ventricular unloading due to the limitations of an administrative database [4,5,7]. The optimal method of left ventricular unloading in this population remains to be defined [4,5]. In light of the high costs of ECMO support, further careful study of complication rates, more standardized reporting and a detailed risk-benefit discussion are needed to optimize patient outcomes. Lastly, the lower rates of angiography and percutaneous coronary intervention in this study are consistent with prior real-world literature that reflects reluctance to perform angiography in higher risk cohorts despite robust guideline recommendations [7].

This study has several limitations that are inherent to the analysis of a large administrative database. The HCUP-NIS attempts to mitigate potential errors by using internal and external quality control measures. The HCUP-NIS database does not provide further granular information on timing of a procedure beyond hospital day, and therefore, the exact sequence of events (i.e., angiography, coronary intervention, ECMO insertion, and timing of complications) cannot be reliably discerned if all happen within the same hospital day. Information on coronary anatomy, successful revascularization, vasoactive medication use and dosing, left ventricular function, peak serum lactate, and hemodynamic variables known to influence outcomes in this population, were unavailable in the HCUP-NIS database. Additionally, because of the non-randomized nature of this study, it is challenging to fully understand the baseline differences in the groups and determine how this has impacted on outcomes. It is conceivable that these patients may have had uniformly high in-hospital mortality in the absence of ECMO support, however, further data are needed from randomized trials. Although in patients with AMI with or without cardiogenic shock, venoarterial ECMO is the most commonly used configuration of ECMO support, it is possible that the ICD-9CM used in this study may refer to the veno-venous ECMO, used for respiratory support (the ICD-10CM codes distinguish these various configurations). Because of the limitations of the administrative coding, this database is unable to distinguish venoarterial and veno-venous configurations. Despite these limitations, this study addresses an important knowledge gap highlighting the national trends and outcomes of in-hospital complications in AMI admissions receiving ECMO support.

In summary, complications in AMI admissions receiving ECMO support have resulted in greater resource utilization during and after the index hospitalization, despite comparable in-hospital mortality. Given the high costs associated with ECMO support, optimal patient and device selection is key in this critically ill population.

## Figures and Tables

**Table 1:** Predictors of in-hospital mortality.

Overall Cohort (*n* = 4608)		Odds Ratio	95% Confidence Interval		*p*
			Lower Limit	Upper Limit	
Any complication		0.89	0.77	1.02	0.10
Age groups (years)	≤75 years	Reference category			
	>75 years	1.54	1.16	2.03	0.003
Sex	Male	Reference category			
	Female	1.21	1.04	1.40	0.01
Race	White	Reference category			
	Non-White ^a^	1.42	1.25	1.63	<0.001
Primary payer	Medicare	Reference category			
	Medicaid	0.59	0.47	0.74	<0.001
	Private	0.49	0.41	0.57	<0.001
	Others ^b^	1.02	0.80	1.29	0.89
Charlson Comorbidity Index	0–3	Reference category			
	4–6	1.00	0.86	1.16	0.99
	≥7	0.97	0.76	1.24	0.82
Hospital teaching status and location	Rural	Reference category			
	Urban non-teaching	0.67	0.37	1.21	0.19
	Urban teaching	1.57	0.91	2.69	0.10
Hospital bed-size	Small	Reference category			
	Medium	0.99	0.66	1.47	0.95
	Large	1.60	1.11	2.31	0.01
Hospital region	Northeast	Reference category			
	Midwest	0.93	0.78	1.11	0.44
	South	0.77	0.66	0.91	0.002
	West	0.98	0.79	1.23	0.89
AMI type	STEMI	Reference category			
	NSTEMI	1.00	0.86	1.15	0.96
Acute organ dysfunction	Respiratory	1.18	1.02	1.35	0.02
	Renal	1.63	1.41	1.88	<0.001
	Hepatic	1.42	1.23	1.65	<0.001
Cardiogenic shock		0.86	0.72	1.01	0.07
Cardiac arrest		1.01	0.89	1.15	0.85
Coronary angiography		1.36	1.17	1.57	<0.001
Percutaneous coronary intervention		0.66	0.57	0.76	<0.001
Pulmonary artery catheterization		0.66	0.54	0.81	<0.001
Second mechanical circulatory support use		0.97	0.85	1.11	0.66
Invasive mechanical ventilation		1.21	1.06	1.39	0.005
Hemodialysis use		1.81	1.39	2.35	<0.001

Legend: ^a^ Black, Hispanic, Asian, Native American, Others; ^b^ Uninsured, No Charge, Others. Abbreviations: AMI: acute myocardial infarction; NSTEMI: non-ST-segment elevation myocardial infarction; STEMI: ST-segment elevation myocardial infarction.

## Supplementary Materials

The following are available online at https://www.mdpi.com/2077-0383/9/3/839/s1, Table S1: Administrative codes, Table S2: Predictors of complications in AMI admissions receiving ECMO.

## Abbreviations

AMI	acute myocardial infarction
ECMO	extracorporeal membrane oxygenation
HCUP	Healthcare Cost and Utilization Project
IABP	intra-aortic balloon pump
ICD-9CM	International Classification of Diseases-9 Clinical Modification
ICD-10CM	International Classification of Diseases-10 Clinical Modification
IQR	interquartile range
NIS	National/Nationwide Inpatient Sample
pLVAD	percutaneous left ventricular assist device
