# Supplementary material for: Complications in Patients with Acute Myocardial Infarction Supported with Extracorporeal Membrane Oxygenation
Source: J Clin Med. 2020 Mar 19;9(3):839. doi: 10.3390/jcm9030839 (PMC7141494; doi:10.3390/jcm9030839)
Supplement: Supplementary file 1 [file jcm-09-00839-s001.pdf]

SUPPLEMENTARY MATERIAL

Complications in Patients with Acute Myocardial Infarction Supported with Extracorporeal Membrane Oxygenation

Saraschandra Vallabhajosyula, Malcolm R Bell, Gurpreet S Sandhu, Allan S Jaffe, David R Holmes, Jr, Gregory W Barsness

Table S1. Administrative codes.

Comorbidity	International Classification of Diseases, 9.0 Clinical Modifications codes
Cardiac arrest	427.5
Cardiogenic shock	785.51
Respiratory failure	518.81, 518.82, 518.85, 786.09, 799.1, 96.7, 96.70, 96.71, 96.72
Hepatic failure	570.0, 572.2, 573.3, 573.4
Coronary angiography	37.22, 37.23, 88.53-88.56
Percutaneous coronary intervention	00.66, 36.01, 36.02, 36.05, 36.06, 36.07, 88.57
Invasive mechanical ventilation	96.7, 96.70, 96.71, 96.72
Hemodialysis	39.95
Vascular complications	904.0, 904.1, 904.2, 904.4, 904.40, 904.41, 904.7, 904.8, 904.9, 998.2, 999.2, 997.2, 997.7, 997.79
Acquired arteriovenous fistula	447.0
Vascular complications requiring surgery	39.31, 39.41, 39.49, 39.52, 39.53, 39.56, 39.57, 39.58, 39.59, 39.79
Lower limb amputation	84.1, 84.10, 84.11, 84.12, 84.13, 84.14, 84.15, 84.16, 84.17, 84.18, 84.19
Post-operative hemorrhage/hematoma	998.11, 998.12, 285.1
Hemolytic anemia	283.0, 283.1, 283.10, 283.11, 283.19, 283.2, 283.9
Thrombocytopenia	287.4, 287.41, 287.49, 287.5, 287.8, 287.9, 289.84
Red blood cell transfusion	99.00, 99.02, 99.03, 99.04
Acute ischemic stroke	433.01, 433.11, 433.21, 433.31, 433.81, 433.91, 436.0, 437.1, 434, 434.0, 434.00, 434.01, 434.1, 434.10, 434.11, 434.9, 434.90, 434.91, 435, 435.0, 435.1, 435.2, 435.3, 435.8, 435.9
Intracranial hemorrhage	430, 431, 432.0, 432.1, 432.9
Acute kidney injury	584, 584.5, 584.6, 584.7, 584.8, 584.9

Table S2. Predictors of complications in AMI admissions receiving ECMO.

Overall cohort (*n* = 4,608)		Odds Ratio	95% Confidence Interval		*P*
			Lower Limit	Upper Limit	
Age groups (years)	≤75 years	Reference category			
	>75 years	1.19	0.92	1.54	0.19
Sex	Male	Reference category			
	Female	1.13	0.98	1.32	0.09
Race	White	Reference category			
	Non-White[a]	1.32	1.15	1.50	<0.001
Primary payer	Medicare	Reference category			
	Medicaid	1.85	1.46	2.34	<0.001
	Private	1.74	1.48	2.04	<0.001
	Others[b]	1.45	1.14	1.84	0.002
Charlson Comorbidity Index	0-3	Reference category			
	4-6	1.93	1.67	2.23	<0.001

	≥ 7	2.11	1.66	2.68	<0.001
Hospital teaching status and location	Rural	Reference category			
	Urban non-teaching	1.39	0.73	2.67	0.32
	Urban teaching	2.20	1.20	4.04	0.011
Hospital bed-size	Small	Reference category			
	Medium	2.13	1.39	3.24	<0.001
	Large	2.26	1.53	3.34	<0.001
Hospital region	Northeast	Reference category			
	Midwest	1.39	1.16	1.66	<0.001
	South	1.21	1.03	1.41	0.02
	West	2.18	1.74	2.73	<0.001
AMI type	STEMI	Reference category			
	NSTEMI	0.97	0.84	1.12	0.65
Acute organ dysfunction	Respiratory	1.25	1.09	1.44	0.001
	Renal	1.34	1.16	1.54	<0.001
	Hepatic	1.03	0.89	1.20	0.67
Cardiogenic shock		1.03	0.87	1.22	0.699
Cardiac arrest		1.12	0.98	1.27	0.10
Coronary angiography		0.88	0.76	1.01	0.07
Percutaneous coronary intervention		0.62	0.53	0.71	<0.001
Pulmonary artery catheterization		1.45	1.16	1.80	0.001
Second mechanical circulatory support use		0.96	0.84	1.10	0.53
Invasive mechanical ventilation		1.51	1.32	1.72	<0.001
Hemodialysis use		3.04	2.29	4.03	<0.001

Legend: [a] Black, Hispanic, Asian, Native American, Others; [b] Uninsured, No Charge, Others. **Abbreviations:** AMI: acute myocardial infarction; ECMO: extracorporeal membrane oxygenation; NSTEMI: non-ST-segment elevation myocardial infarction; STEMI: ST-segment elevation myocardial infarction.
